# Supplementary material for: Vitamin D Modified DSS-Induced Colitis in Mice via STING Signaling Pathway
Source: Biology (Basel). 2025 Jun 18;14(6):715. doi: 10.3390/biology14060715 (PMC12190092; doi:10.3390/biology14060715)
Supplement: Supplementary file 1 [file biology-14-00715-s001.zip › Supplementary Materials S1: 16s-report of gut microbiota/Groups/FAPROTAX/Markers/FAPROTAX.Groups.sig.boxplot.pdf]

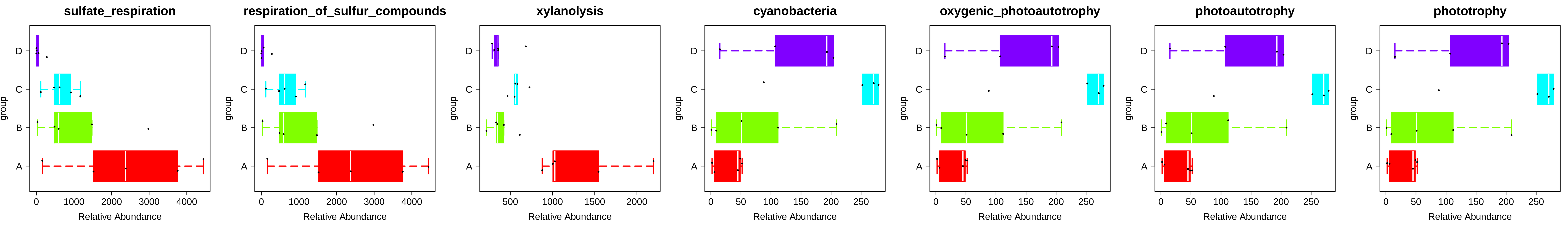
sulfate_respiration
respiration_of_sulfur_compounds
xylanolysis
cyanobacteria
oxygenic_photoautotrophy
photoautotrophy
phototrophy
group
D
C
B
A
Relative Abundance
0 1000 2000 3000 4000
500 1000 1500 2000
0 50 100 150 200 250
